# Decreased Free Water Clearance Is Associated with Worse Respiratory Outcomes in Premature Infants

**DOI:** 10.1371/journal.pone.0016995

**Published:** 2011-02-10

**Authors:** Tuomo Vuohelainen, Riitta Ojala, Anita Virtanen, Päivi Korhonen, Tiina Luukkaala, Päivi Holm, Outi Tammela

**Affiliations:** 1 Paediatric Research Centre, University of Tampere and Tampere University Hospital, University of Tampere, Tampere, Finland; 2 Department of Paediatrics, Tampere University Hospital, Tampere, Finland; 3 Science Center, Pirkanmaa Hospital District, Tampere, Finland; 4 Centre for Laboratory Medicine, Tampere University Hospital, Tampere, Finland; Cincinnati Children's Hospital Medical Center, United States of America

## Abstract

**Objective:**

The goal was to elucidate predictors of decreased free water clearance (DFWC) in very low birth weight (VLBW) infants. We hypothesized that DFWC and fluid retention are linked to the severity of pulmonary problems and prolonged respiratory support, especially to nCPAP treatment.

**Methods:**

The investigation was carried out at Tampere University Hospital between 2001 and 2006. The study population comprised 74 VLBW infants born at 29.21 (24.57–34.14) weeks of gestation. Median birth weight was 1175 (575–1490) grams. We measured plasma and urine osmolality and 24-hour urine volume to calculate free water clearance (FWC) for each infant. If FWC was less than 30 ml/kg/day the infant was classified as having DFWC.

**Results:**

There were 38 (51.4%) infants with DFWC in the study population. The median duration of the observed DFT period was 14 (4–44) days. The gestational age at birth was lower for DFWC infants compared to infants with normal FWC (NFWC), 28.29 (24.57–32.86) vs. 30.00 (25.57–34.14) weeks (p = 0.001). DFWC infants also needed longer ventilator treatment, 2 (0–23) vs. 0.50 (0–23) days (p = 0.046), nCPAP treatment 30 (0–100) vs. 3 (0–41) days (p<0.0001) and longer oxygen supplementation 47 (0–163) vs. 22 (0–74) days (p = 0.011) than NFWC infants. All values presented here are medians with ranges.

**Conclusions:**

DFWC appears to be frequently connected with exacerbation and prolongation of pulmonary problems in VLBW infants. Cautious fluid administration seems to be indicated in VLBW infants with prolonged respiratory problems and DFWC.

## Introduction

Infants with bronchopulmonary dysplasia (BPD) appear to suffer transiently from decreased free water clearance (DFWC) on certain occasions [1], [2]. This is manifested by systemic and pulmonary edema, oliguria, hyponatremia and hypertension, often currently managed with diuretics [3]. Intervention by fluid restriction has also been recommended [4].

Earlier studies on fluid balance have concentrated on fluid management during the first days after the birth and have shown that restriction of fluid intake decreases the mortality and morbidity in preterm infants [5], [6]. It is, however, not clear, which infants might get benefit from fluid restriction, and how long it is needed in such cases.

An inappropriate arginine vasopressin (AVP) hypersecretion has emerged as one of the factors underlying the tendency to fluid retention in BPD infants [1], [2]. AVP is a neurophyseal peptide hormone which among other functions regulates fluid balance. In our two earlier studies we showed AVP hypersecretion to induce transient fluid retention in response to prolonged or otherwise stressful birth in healthy term infants [7], [8]. This stress response appears to be purposeful and is evidently physiological, unlike the fluid retention with VLBW infants observed in our present study.

Continuous positive airway pressure (CPAP) treatment increases AVP secretion, reduces the secretion of natriuretic peptides and imposes fluid retention [9], [10], [11], [12], [13]. The secretion of these fluid balance homeostasis-regulating hormones is mutually intertwined. AVP stimulates a release of natriuretic peptides which subsequently reduce AVP secretion through a negative feed-back loop [14]. Natriuretic peptides are cardiac hormones which modulate extracellular fluid volume and blood pressure by stimulating natriuresis, vasodilation and inhibition of AVP release in response to pressure or volume overload [14], [15], [16]. Hence at least partially an iatrogenic imbalance in this homeostatic system with the consequent fluid retention may exacerbate pulmonary problems and further prolong need for the respiratory support of BPD infants, as indeed suggested almost thirty years ago [17]. A recent study with an animal model lends support to these hypotheses [18].

In our clinical practice we have noted a tendency to fluid retention in VLBW infants, linked to prolongation of pulmonary problems and especially BPD. During past decades this phenomenon has been unfortunately disregarded in neonatological research and also in clinical work. Our aim here was to identify predictors of DFWC in a population of VLBW infants. We hypothesized that the fluid retention tendency is linked to pulmonary problems and a prolonged need of respiratory support, especially nasal CPAP (nCPAP) treatment. As DFWC can be regarded as an indirect indicator of AVP activity, we chose this more convenient means in evaluating fluid balance.

## Methods

The present investigation was carried out at Tampere University Hospital between the years 2001 and 2006. The Ethics Committee of the Pirkanmaa Hospital District approved the study protocol and informed written consent was obtained from the parents participating.

Infants of birth weight less than 1500 g without any major congenital malformations or grade IV intraventricular hemorrhage were enrolled. Infants were considered small for gestational age (SGA) if they were in the lowest 10th percentile of birth weight and appropriate for gestational age if they were above 10th percentile for each gestational week stratified by infant gender [19].

Starting from the calendar age of about one week, free water clearance (FWC) and fluid input (total enteral and/or parenteral 24-hour fluid intake)/output (urine output) ratio were determined and weight, need of oxygen (O_2_) supplementation, assisted ventilation and corticosteroid use at that time-point were recorded. Thereafter, the corresponding data were collected sequentially at two weeks' intervals until 36 weeks' corrected gestational age or until the end of O_2_ supplementation, or until FWC normalized. Diuretics or indomethacine were not administered on the study day. The 24-hour urine volume was determined by weighing diapers. Urine osmolality (U-Osm) was determined from a single bag urine sample as closely simultaneous as possible with the blood sample for serum osmolality (S-Osm) determination. From these data the FWC was calculated by the following formula:
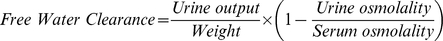



If FWC was less than 30 ml/kg/day the infant was classified as having DFWC [1]. This mode of assessment was considered reliable in that daily parenteral fluids were administered as continuous infusions and daily enteral feedings in evenly divided volumes at three hour's intervals.

Prophylactic surfactant was administered to all infants born at gestational ages less than 28 weeks, and at gestational ages 28–30 weeks if the mother had not received antenatal glucocorticoids and/or the infant needed delivery room intubation. Infants of gestational ages 30 weeks or more received surfactant as rescue therapy for RDS at the discretion of the attending neonatologist.

The diagnosis of BPD was established at the calendar age of 28 days or at a corrected gestational age of 36 weeks in infants [20], [21], [22]. The severity of BPD was graded according to the need for O_2_ [22]. An O_2_ saturation target between 88 and 94% was used in the unit. All chest radiographs, obtained according to clinical indications were assessed by a pediatric radiologist. BPD findings were classified as hazy to opaque or bubbly chest images [23].

Between 2001 and 2005 U-Osm and S-Osm were determined using a Cryomatic 3C2 (Advanced Instruments inc., Massachusetts, USA) analyzer by a method based on freezing point measurement. After October 2005 these analyses were made by a Knauer A0300 (Knauer, Berlin, Germany) analyzer, likewise based on freezing point measurement.

### Statistics

It was assumed that an infant with pulmonary problems involving a need for O_2_ supplementation, nCPAP or mechanical ventilation would have 30% lower free water clearance (FWC) than an infant without such problems at 28 days of age. Based on this assumption a preliminary power analysis was made and an appropriate statistical power of 80% and a statistical significance of *p<0.05* were estimated to be achieved with 40 infants per group. All data were analyzed using SPSS Statistics Release 17.0.0 (SPSS Inc., Chicago, IL, USA). Continuous data were analyzed using Spearman's rank correlation, Mann-Whitney U-test or Kruskal Wallis test, and discrete data were analyzed by the Pearson chi-squared test or Fisher's exact test. Results are expressed as occurrences (%) or median (range) values. Kaplan-Meier survival curves were analyzed using the Log Rank Cox-Mantel test. The first survival analysis addressed the cumulative occurrence of normal FWC between nCPAP-treated infants and those who managed without it. In the second survival analysis the cumulative occurrence of supplemental O_2_ need was compared between infants with normal and abnormal fluid tolerances. Factors associated with the infants' decreased FWC were analyzed by backward-stepwise logistic regression, with *p<0.05* and a 95% confidence interval (CI) to indicate statistical significance. Covariates included in the model were sex, gestational age at birth, birth weight, surfactant treatment, BPD diagnosis and durations of ventilator treatment, nCPAP treatment and supplemental O_2_.

## Results

The study population comprised 74 very low birth weight (VLBW) infants of median 29.2 (range 24.6–34.1) weeks' gestation. The median birth weight was 1175 (range 575–1490) grams.

DFWC was detected in 38 (51.4%) infants (Table 1). The median corrected gestational age at first DFWC finding was 31.0 (27.4–36.3) weeks and the median calendar age at this point was 21 (range 6–48) days. In 19 of these, FWC was first normal and changed to DFWC in further measurements. At the time of the first DFWC 25 (65.8%) of the infants received nCPAP treatment, 5 (13.2%) needed supplemental O_2_ and 8 did not need any of these. None of the DFWC infants received mechanical ventilation at this point.

**Table 1:** Characteristics of 74 study infants.

	DFWC		NFWC		*p*
Number of infants, n (%)	38	(51.4)	36	(48.6)	
FWC measurements, Md (Range)	4	(1–6)	2	(1–6)	<0.001
Boys, n (%)	24	(63.2)	22	(61.1)	1.000
Gestational age at birth (wk), Md (Range)	28.3	(24.6–32.9)	30.0	(25.6–34.1)	0.001
Birth weight (g), Md (Range)	1050	(625–1490)	1280	(575–1490)	0.007
SGA, n (%)	6	(15.8)	12	(33.3)	0.106
Prenatal glucocorticoid, n (%)	34	(89.5)	31	(86.1)	0.618
Surfactant treatment, n (%)	29	(76.3)	18	(50.0)	0.029
BPD, n (%)	25	(65.8)	11	(32.4)	0.009
Moderate or severe BPD, n (%)	16	(42.1)	7	(19.4)	0.047
Assisted ventilation (days), Md (Range)	2	(0–23)	0.5	(0–23)	0.046
nCPAP (days), Md (Range)	30	(0–100)	3	(0–41)	<0.001
O2 supplementation (days), Md (Range)	47	(0–163)	22	(0–74)	0.011
Hospitalization (days), Md (Range)	72	(26–15)	53	(22–262)	<0.001
Diuretic treatment, n (%)	8	(21.1)	5	(13.9)	0.545
Indomethacin treatment, n (%)	0	(0)	2	(5.6)	0.233
Died, n (%)	1	1(2.6)	1	(2.8)	1.000

Results are expressed as occurrences (%) or median (range) values with statistical significance (*p*)*.

*Differences between DFWC and NFWC were tested by Mann-Whitney test or by Pearson chi-square or Fisher's exact test.

FWC normalized in 25 (65.8%) of the DFWC infants. The remaining 13 were transferred to their regional hospitals, or their clinical condition otherwise improved and further FWC measurements were not undertaken. The median duration of the observed DFWC period was 14 (range 4–44) days. At the end of this period the median corrected gestational age was 34.1 (range 28.4–38.3) weeks and the median calendar age 40 (range 14–69) days. At this point 12 (48.0%) infants still needed nCPAP treatment and three (12.0%) supplemental O_2_.

**Figure 1:**
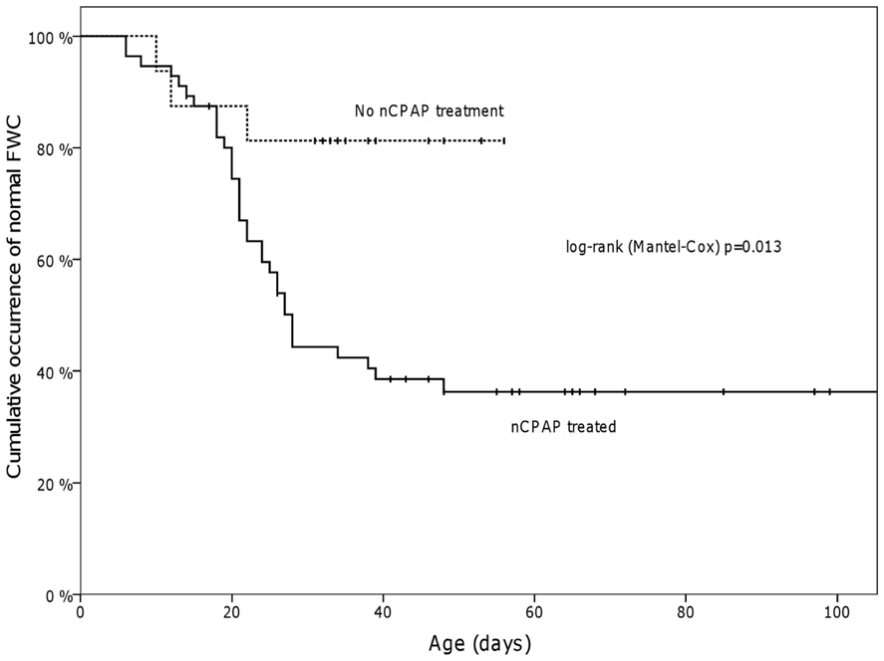
The cumulative occurrence of normal FWC between nCPAP-treated infants and infants who managed without it.

**Figure 2:**
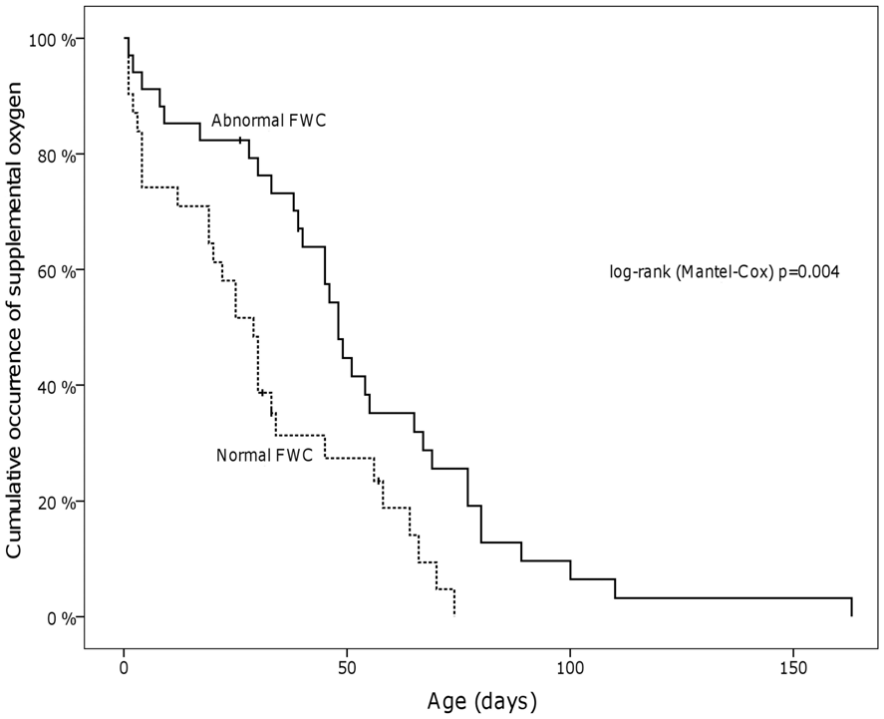
The cumulative occurrence of supplemental oxygen compared between infants with normal and abnormal fluid tolerances.

In survival analysis we also observed a significant difference (p = 0.013) in DFWC occurrence between nCPAP-treated infants and those who managed without it [Figure 1]. The need for supplemental O_2_ lasted significantly longer in DFWC infants than in NFWC infants (p = 0.004) [Figure 2].

Eighteen infants were treated with budesonide inhalations when the first decreased FWC was determined. None of these infants received systemic glucocorticoid treatment at this point. The budesonide treatment was not associated with the duration of DFWC nor with the need for respiratory support at the end of the DFWC period.

In backward logistic regression analysis only the duration of nCPAP treatment [p = 0.001; odds ratio 1.059; 95% CI, 1.024–1.095] predicted DFWC statistically significantly.

To establish occasion-related variables associated with low FWC in DFWC infants we analyzed the fluid balance measurements closest to 21 day's postnatal age (Table 2). This time-point (D21) was selected according to the median age at detection of DFWC. At D21 there were 27 (36.5%) infants with DFWC (DFWC21), two (2.7%) infants had already recovered from it and 9 (12.2%) developed DFWC later, as shown in Figure 1. Since these 11 infants did not evince DFWC at D21 they were excluded from this analysis. Corresponding measurements at D21 (NFWC21) in study infants with normal FWC were analyzed as control data.

**Table 2:** Fluid balance data at postnatal age of 21 days in infants with abnormal and normal free water clearance.

	DFWC21 (n = 27)		NFWC21 (n = 36)		*p*
Boys, n (%)	18	(66.7)	22	(61.1)	0.793
Gestational age at birth (wk), Md (Range)	27.7	(24.6–32.9)	30.0	(25.6–34.1)	0.001
Birth weight (g), Md (Range)	1005	(625–1490)	1280	(575–1490)	0.003
SGA, n (%)	4	(14.8)	12	(33.3)	0.095
Weight at D21 (g), Md (Range)	1141	(705–2045)	1565	(792–2120)	0.005
Free water clearance (g/kg/d), Md (Range)	−4.9	(−213.8–28.5)	57.1	(34.3–92.9)	<0.001
Serum osmolality (mOsm/L), Md (Range)	272	(256–296)	276	(260–295)	0.140
Urine osmolality (mOsm/L), Md (Range)	280	(189–701)	141.5	(75–185)	<0.001
Fluid intake (ml/kg/d), Md (Range)	171.8	(117.1–195.9)	176.6	(134.1–229.1)	0.013
Urine volume (ml/kg/d)	99.0	(39.2–241.7)	120.0	(60.9–177.9)	0.004
Intake/Output ratio, Md (Range)	1.61	(0.62–2.65)	1.47	(0.94–3.10)	0.092
Ongoing nCPAP treatment, n (%)	17	(63.0)	12	(33.3)	0.024
Before D21 duration, Md (Range)					
nCPAP treatment	12	0–22)	3	(0–21)	0.025
Assisted ventilation	20	(0–28)	7	(0–24)	0.011

Results are expressed as occurrences (%) or median (range) values with statistical significance (*p*)*.

*Differences between DFWC21 and NFWC21 were tested by Mann-Whitney test or by Pearson chi-square or Fisher's exact test.

A significant negative correlation was observed between FWC and the duration of nCPAP treatment before D21 (r = −0.335, p = 0.007). BPD findings in chest X-ray images obtained closest to D21 were associated with decreased FWC 12.3 (−213.8–92.9) vs. 56.2 (−9.0–89.9) ml/kg/day (p<0.001). Since all chest X-ray images were taken on clinical indications, we here reviewed the radiographs closest to D21, the median difference being −3 (−26–17) days.

## Discussion

Earlier clinical studies have concentrated mainly on fluid management of premature infants' early days or first week [5], [6]. In our work we focused on long-term fluid management and used FWC to evaluate and monitor fluid balance and retention.

The results of this study suggest that low FWC is associated with pulmonary problems in VLBW infants. As these problems decrease free water clearance also will eventually normalize. This coincides with our clinical experiences and supports earlier hypotheses regarding poor tolerance to fluids in such infants. Nonetheless, the precise etiology and the underlying pathophysiology of decreased fluid tolerance in this patient group remain unclear.

There was a statistically significant difference between the median fluid intake at D21 between DFWC21 and NFWC21 infants. The difference between the two figures was notably small, about five ml/kg/day, and therefore not clinically significant. The aim was not to blind the clinicians for the FWC results, because infants with DFWC get benefit from restricted fluid administration. Bias due to this is, however, very unlikely, because same guidelines for weaning from respiratory support and oxygen supplementation were followed in the treatment of all infants in the unit. If any bias would exist, it would not change the main results, because fluid restriction would more probably alleviate than increase the need of respiratory support and oxygen supplementation. The urine volumes were lower and U-Osm higher in the DFWC group compared to NFWC cases, but conversely, S-Osm tended to be lower in the infants with DFWC than in NFWC cases. The differences cannot thus be explained by dehydration in the patients with DFWC.

Gestational age at birth and birth weight were lower among DFWC infants. The greater immaturity of DFWC infants obviously predicted higher frequencies of surfactant treatment and BPD in this group, and DFWC infants accordingly needed longer periods of ventilator treatment, supplemental O_2_ and hospitalization than NFWC infants. Immaturity is nonetheless unlikely to be the sole predictor of DFWC problems, since there was wide variance in corrected gestational age at the first decreased FWC. This reasoning is further substantiated by the result of the logistic regression analysis and the fact that in half of the DFWC cases the FWC was normal in the first measurement.

We observed a marked association between the occurrence of DFWC and the duration of nCPAP treatment, this again lending support to earlier findings [9], [10], [11], [12], [13], [14], [15]. As shown in Figure 1, nCPAP-treated infants developed DFWC more frequently than infants who managed without it.

Mechanical ventilation and positive airway pressure induce fluid retention through multiple intertwined mechanisms. These include positive end-expiratory pressure, raising intrathoracic and hence inferior vena cava pressure, and both in turn stimulate AVP secretion [9], [10], [15]. Secondly, positive airway pressure has also been shown to reduce ANP secretion by reducing atrial distension [11], [12]. Finally, mechanical ventilation and CPAP treatment also cause fluid retention without any changes in AVP or renin activity, probably in consequence of diminished cardiac output [13]. Previously Hazinski and associates measured AVP levels in infants with chronic BPD and DFWC [1]. Direct measurement of AVP activity is, however, poorly suitable for clinical work in that it is expensive and relatively time-consuming, as we have experienced with AVP radioimmunoassays [7].

Imbalance in fluid homeostasis in DFWC infants seems to be caused by inappropriate AVP hypersecretion due various non-osmotic stimuli from stress, hypoxia and also directly from respiratory support, especially from positive airway pressure as previously described [1], [2], [9], [10], [11], [12], [13], [15], [16], [20], [24]. Our present results lend strong support to these previous findings. Also the analysis at D21 would advocate such an interpretation to an even greater extent. The long duration of nCPAP treatment and respiratory support were strong predictors of DFWC. Also the majority of DFWC infants were treated with nCPAP at the time of the first decreased FWC measurement.

DFWC infants required O_2_ supplementation longer than NFWC infants [Figure 2]. As we hypothesized, fluid retention causes pulmonary edema and further impairs DFWC infants' oxygenation, and will prolong and aggravate BPD. Also, as Hazinski suggested based on earlier studies of BPD and status asthmaticus [25]–[27], reduced pulmonary functional residual capacity and increased alveolar dead space lead to increased intra thoracic pressure and decreased venous return to the left atrium [1]. This increases AVP secretion and causes poor tolerance to fluids in BPD patients even without nCPAP treatment [1]. The same phenomenon was noted in chest radiographs taken at D21. These revealed a significant association between low FWC and abnormal findings related to BPD. DFWC seems thus to be a vicious cycle which needs to be recognized. In light of this, excessive fluid administration needs to be avoided in VLBW infants with prolonged pulmonary problems and in such cases fluid restriction might be more beneficial mode of treatment than diuretics. In this way, the side-effects of diuretics can also be avoided. During the fluid restriction period it is paramount to ascertain that these infants attain adequate caloric intake in order to thrive.

### Conclusions

The present findings suggest that DFWC is markedly associated with exacerbation and prolongation of pulmonary problems and the need for nCPAP treatment in VLBW infants. As also shown in earlier studies, the hypersecretion of AVP stimulated by various circulatory and pulmonary mechanical factors would appear to induce fluid retention. In establishing, whether the infant's fluid intake needs to be cautious, fluid tolerance can be assessed by determining FWC.
